# Dried fluid spots for peste des petits ruminants virus load evaluation allowing for non-invasive diagnosis and genotyping

**DOI:** 10.1186/s12917-014-0247-y

**Published:** 2014-10-11

**Authors:** Ataur Rahman Bhuiyan, Emdadul Haque Chowdhury, Olivier Kwiatek, Rokshana Parvin, Mushfiqur M Rahman, Mohammad R Islam, Emmanuel Albina, Geneviève Libeau

**Affiliations:** Department of Livestock Services, Dhaka, Bangladesh; Department of Pathology, Bangladesh Agricultural University (BAU), 2202 Mymensingh, Bangladesh; CIRAD, UMR CMAEE, F-34398 Montpellier, France; INRA, UMR 1309 CMAEE, F-34398 Montpellier, France; CIRAD, UMR CMAEE, F-97170 Petit-Bourg, Guadeloupe France

**Keywords:** Peste des petits ruminants virus, Filter paper, Genotyping, Field samples

## Abstract

**Background:**

Active surveillance of peste des petits ruminants (PPR) should ease prevention and control of this disease widely present across Africa, Middle East, central and southern Asia. PPR is now present in Turkey at the gateway to the European Union. In Bangladesh, the diagnosis and genotyping of PPR virus (PPRV) may be hampered by inadequate infrastructures and by lack of proper clinical material, which is often not preserved under cold chain up to laboratories. It has been shown previously that Whatman® 3MM filter paper (GE Healthcare, France) preserves the nucleic acid of PPRV for at least 3 months at 32°C.

**Results:**

In this study, we demonstrate the performances of filter papers for archiving RNA from local PPRV field isolates for further molecular detection and genotyping of PPRV, at −70°C combined with ambient temperature, for periods up to 16 months. PPR-suspected live animals were sampled and their blood and nasal swabs were applied on filter papers then air dried. Immediately after field sampling, RT-PCR amplifying a 448-bp fragment of the F gene appeared positive for both blood and nasal swabs when animals were in febrile stage and only nasal swabs were detected positive in non-febrile stage. Those tested positive were monitored by RT-PCR up to 10 months by storage at −70°C. At 16 months, using real time RT-PCR adapted to amplify the N gene from filter paper, high viral loads could still be detected (~2 x 10^7^ copy numbers), essentially from nasal samples. The material was successfully sequenced and a Bayesian phylogenetic reconstruction achieved adequate resolution to establish temporal relationships within or between the geographical clusters of the PPRV strains.

**Conclusions:**

This clearly reveals the excellent capacity of filter papers to store genetic material that can be sampled using a non-invasive approach.

## Background

Peste des petits ruminants virus (PPRV), a member of the genus *Morbillivirus* in the family *Paramyxoviridae* [1], is the causative agent of peste des petits ruminants (PPR), an acute viral disease of goats and sheep characterized by fever, erosive stomatitis, conjunctivitis, diarrhoea, pneumonia and death [2,3]. This disease is characterized by viraemia that can develop 2–3 days after infection and 1–2 days before the first clinical sign appears and that extends until the animal develops an immune response. During the initial viraemic stage, almost no clinical signs occur except fever [4]. In Bangladesh, PPR was confirmed from an isolate of 1993 [5] and has been reported in goats since then [6]. The disease causes severe losses to small ruminant production and is presently considered as one of the major threats to about 22 million small ruminants in Bangladesh, where mortality may reach up to 100% in an outbreak. Until recently, no laboratory facilities were available to diagnose the disease in that country. First generation tests such as virus neutralization and the agar immunodiffusion tests are either time-consuming, labour intensive to perform, or nonspecific. In the past ten years, developments in molecular biology have improved diagnosis and epidemiological knowledge of PPR. RT-PCR is an essential tool for investigating the epidemiology of the disease and phylogeny of the virus [5,7-10]. In addition, Taqman real time PCR (qRT-PCR) speeds up PPRV diagnosis [11-13]. The genotyping of PPRV reveals four geographically separated lineages. Diversity study is increasingly recognized as a powerful approach in epidemiological and evolutionary research. Molecular diagnosis or virus isolation from the infected materials, however, requires a cooling chain (−20°C) that is difficult to achieve in field sampling. A significant breakthrough was achieved through filter papers for the diagnosis of diseases, genotyping and sero-surveillance. Filter papers have been widely used to store samples [14-16] for molecular studies of viruses after nucleic acid extraction [17] or without extraction [18,14,19,20].

Current methods require the handling of animals for sampling blood or post-mortem tissues. Although with some expertise this is relatively straightforward, it requires equipment for sample collection, processing, and storage, which may be lacking in rural areas, especially the most remote ones. Furthermore, alternatives to venous blood collection are preferred by the poor herders. Ocular and nasal swabs are stress less and simple to collect, and swab samples have proved to provide PPR viral RNA [21,10]. In the present study we therefore compared the ability of the Whatman® 3MM filter to archive PPRV from nasal swabs and from blood samples when collected in field conditions. We described further molecular detection of PPRV from the paper matrix by direct amplification methods and in addition, using Bayesian analysis, we studied the evolutionary relationship of the viral strains involved in outbreaks that occurred in the Southern Asian region.

## Results

### Outbreaks and status of the sampled animals

Three outbreaks were investigated in different locations of Dhaka and Narayangonj, Bengladesh, between March and May 2008. All the goats of three traditional farms were black Bengal with different age groups. Goat numbers were 27, 8 and 54, respectively in the three farms. In these farms morbidity (and mortality) were 87% (41%), 100% (62%) and 96% (30%). Animals showed clinical symptoms like high temperature, which was accompanied by watery discharges (nasal, ocular and oral), conjunctivitis, erosive stomatitis, diarrhoea, pneumonia, abortion of pregnant animals and death. Eighteen animals were collected in different stages of the disease from the three farms. A group of 12 goats was in the acute stage of disease with fever (40-42°C) along with serous ocular and nasal secretion, 2 goats had almost normal body temperature (38-39°C) but with profuse nasal secretion and diarrhoea, while another 4 goats were at convalescent stages with only moderate diarrhoea and mucoid nasal secretion. Each animal was sampled by using a lymph node suspension, a nasal swab, and a blood sample to smear filter papers, further tested by RT-PCR designed to amplify the F gene directly from filter paper. This preliminary study showed, after immediate sampling on filter paper, that amplicons were obtained for both nasal swabs and blood samples but not for the lymph node suspension. Fifteen out of the 18 animals (83%) tested positive using any of the two samples. For the animals (12) that were in feverish conditions, no significant difference in sensitivity was observed with both types of samples used. For the two other groups of animals (6) that did not exhibit fever, the positives were detected only through the nasal sample. In summary, among the three groups of animals, 14 were found positive using the nasal swabs and 12 were found positive using the blood samples (Table 1).

**Table 1:** **Sensitivity of the RT-PCR method on nasal swab- and blood-smeared filter paper according to the stage of the disease**

**Sampled goats**	**Number of animals tested**	**Samples positive using**	
		Nasal swab	Blood
Goat with fever and profuse nasal secretion	12	11	12
Goat with nasal secretion and diarrhoea, no fever	2	2	-
Goat with diarrhoea, scanty nasal secretion, no fever	4	1	-
Total	18	14	12

### Preservation of viral RNA from deep-frozen filter papers with nasal and blood samples

Six pairs of blood and nasal swabs sampled on filter paper were constituted from animals that were found positive on both samples after immediate sampling using RT-PCR on the F gene. Using this technique, the samples stored at −70°C were monitored each month to check the stability of viral RNA. One out of six blood-soaked papers and four out of six nasal-smear filter papers were found positive after up to 10 months of storage. After an additional 6 months of storage at −70°C, and one week at ambient temperature for shipment between the two laboratories (DPBAU-CIRAD), these samples were tested by qRT-PCR designed to quantify the N gene directly from filter paper. qRT-PCR detected the virus in the previous five positive samples that originated from nasal swab smears and blood, with remarkable viral loads. Three nasal swabs had individual threshold (C*t*) values <17, and another a moderate viral load, C*t* <30, while the blood sample remained over 35 Ct. Estimated viral loads expressed in copy numbers in nasal samples were quite high (~2 × 10^7^) (Table 2).

**Table 2:** **Stability of nasal swab- and blood-smeared filter papers stored up to 16 months at – 70** **°** **C, tested by RT-PCR and qRT-PCR**

	**RT-PCR**											**qRT-PCR**	
	**Sampling dates**												
**Sample**	**May/08**	**June/08**	**July/08**	**Aug/08**	**Sep/08**	**Oct/08**	**Nov/08**	**Dec/08**	**Jan/09**	**Feb/09**	**Mar/09**	**Oct/09**	
												**Ct values**	**Copy number**
Blood	+	+	+	+	+	-	-	-	-	-	-	No	No
Nasal	+	+	+	+	+	+	+	+	+	+	+	16.56	1.67 × 10 ^7^
Blood	+	+	+	+	+	+	+	-	-	-	-	No	No
Nasal	+	+	+	+	+	+	+	+	+	+	-	No	No
Blood	+	+	+	+	+	-	-	-	-	-	-	No	No
Nasal	+	+	+	+	+	+	+	+	+	+	+	16.5	1.75 × 10^7^
Blood	+	+	+	+	+	+	+	+	+	+	-	35.62	1.27 × 10^1^
Nasal	+	+	+	+	+	+	+	+	+	+	+	16.35	1.95 × 10^7^
Blood	+	+	+	-	-	-	-	-	-	-	-	No	No
Nasal	+	+	+	+	+	+	+	+	+	+	-	No	No
Blood	+	+	+	+	+	+	+	+	+	-	-	No	No
Nasal	+	+	+	+	+	+	+	+	+	+	+	29.84	9.08 × 10^2^

+Positive sample by RT-PCR.

-Negative sample by RT-PCR.

C*t*Threshold crossing values.

### Genotyping of the virus collected on Whatman® 3MM filter papers

**Figure 1:**
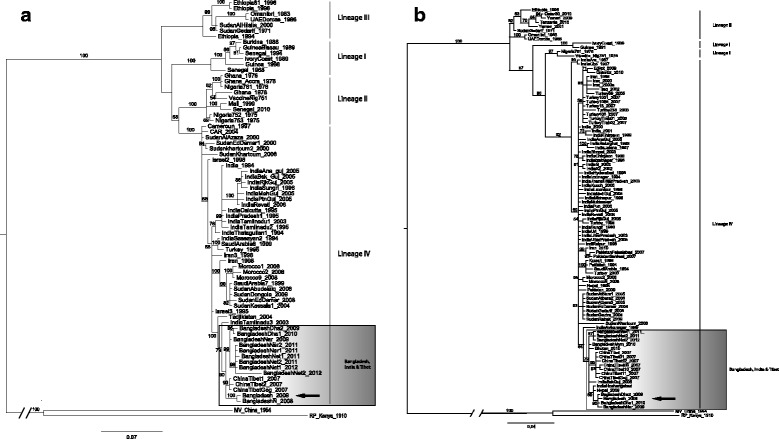
**Bayesian inference of PPRV strains (Bangladesh_2009 and published strains) based on the partial nucleotide sequence of the N protein gene (a) and F protein gene (b).** PPR strains from the four different lineages were selected from available published sequences in GenBank. Branches are identified with black lines according to the four PPRV lineages: lineage I, II, III, or lineage IV. Bangladesh sample (Bangladesh_2009) is arrowed in lineage IV. The year of collection is mentioned for each strain. Branch values correspond to posterior probabilities and the scale bar represents the amounts of expected evolutionary changes per site. The trees were rooted by two out-groups, measles and rinderpest virus collected from GenBank (FJ416068 and X98291, respectively).

Bayesian phylogenetic analysis with a molecular clock assumption was applied to the partial nucleotide sequence of the N protein gene (Figure 1a) and F protein gene (Figure 1b). The main objective of this analysis was to determine the evolutionary relationship among strains of PPRV. Both genes yielded identical tree topology matching in addition previously assigned lineages obtained by Neighbour Joining analysis that used the same portions of these genes [22]. Relationships between the four lineages were supported by Bayesian posterior probabilities between 60% and 100%. Where the strains analysed were more numerous, particularly in lineage IV, the phylogeny was resolved into clusters corresponding to strains with the same regional localisation. Indeed, lineage IV is the most geographically diverse, with sequences from different continents and sub-continents: India, China, Near-East and Africa. In both the F and N phylogenetic trees, the Bangladesh_2009 strain fell into lineage IV and was closely related to Bhutan, China-Tibet, India, Tajikistan and Nepal according to the strains analysed in these trees, forming a Southern Asian clade. In the N tree, the phylogenetic position of the Bangladesh_2009 strain was the MRCA (most recent common ancestor) of the most recent isolates in Bangladesh. In addition, the most ancestral branch for the Southern Asian clade was represented by Tajikistan and Indian strains.

## Discussion

In this experiment we have demonstrated that Whatman® 3MM filter papers offers the advantages of a stable specimen matrix as it can be successfully used to preserve PPRV viral RNA for extended periods of time at −70°C and 7 days at ambient temperature. In addition by comparing dried spots on filter paper made of whole blood and nasal swabs from animals sampled in the field at different clinical stages of the disease, we demonstrated that nasal samples hold promise as an affordable and practical alternative specimen source to whole blood for PPRV viral load determination and further identification.

In outbreaks, dried spots on filter paper made of whole blood or nasal swabs taken from the same animal, appeared positive when animals were in the febrile phase while in the non-febrile phase, only nasal swabs yielded positive detection. Discrepancy between blood and nasal secretions is due to the relatively short period during which the virus replicates in the blood stream until immune response occurs. The early viraemic stage accompanies the febrile condition, which usually persists 2–3 days [4]. The virus localizes concomitantly in the respiratory tract and associated lymph nodes but persists longer, as evidenced by oral and nasal secretions in association with the other symptoms commonly observed when the temperature declines. The width of the diagnostic window in the case of highly pathogenic viruses, like the ones involved in the three outbreaks, was described in two previous experimental infections of goats. Virus was detected from ocular samples by RT-PCR as early as day 3 after inoculation and onwards, before the goats showed clinical signs [23]. In blood, PPRV RNA was limited in time to only day 4 to day 8 after the oronasal challenges [20].

We also demonstrated that the Whatman® 3MM paper retains RNA from nasal swabs for at least 10 months at −70°C while RNA from blood is preserved up to 9 months. The viral load was measured using a direct RT-conventional or real-time PCRs without previous nucleic acid extraction. RT-PCR amplifying a 448-bp fragment of the F gene was used for immediate characterization and stability study of the viral RNA. Then the qRT-PCR targeting the N gene, was used at 16 months after collection. Thus, taken together with the excellent capacity of filter papers to store genetic material, these results give a clear advantage to the nasal over the blood sample for PPRV detection on the long-term for future molecular analysis. In addition, these two features make the filter papers an economical option for veterinary services and laboratories of emerging countries.

Several studies have been performed on the stability of RNA trapped on Whatman 3MM filter papers. Rapid and efficient immobilization of virus as well as high efficiency in RT-PCR was reported for measles virus, another member of the *Morbillivirus* genus [24]. The use of filter paper to transport and recover MV RNA has been described for blood samples [25,24,26]. Drawbacks were however demonstrated in the detection of viral RNA in the blood sampling because of the presence of specific anti-measles virus. An alternative was proposed to collect oral fluid onto filter papers for the detection of MV by RT-PCR [27]. In these studies only environmental temperatures of storage were tested to reproduce the tropical conditions. None of them could preserve the virus more than two to three months, due to suboptimal conditions of desiccation of the virus on the filter paper. Indeed, even in the lyophilized form, the virus loses nearly one log in 144 hours at 37°C and in 120 hours at 45°C [28]. Non-freezing temperatures seem less adequate for middle-term stability in view of subsequent analysis.

In addition, this study demonstrated the possibility to perform sequencing and phylogenetic reconstructions from viral nucleic acid successfully amplified by PCR assay from the Whatman® 3MM matrix. A general rule until recently [29] was geographical clustering of PPRV strains. Though less commonly used, Bayesian analysis is able to determine the evolutionary relationship among strains. Using this approach and with reference to previous observations [5,7,9], phylogenetic reconstructions based on the F and of the N genes showed that Bangladesh_2009 isolate clustered in lineage IV. The improvement of Bayesian analysis is to allow statistically more reliable posterior probabilities of branches than bootstrap probabilities derived from other methods [30]. In addition, the Bayesian method achieves adequate resolution for temporal relationships between geographical distant strains. This is of utmost importance in context of worldwide trade, leading to intensification of animal movements. This method linked to very simple means of sampling and a sensitive tool, the Whatman® 3MM matrix, thus demonstrates its utility and compatibility with genetic characterization of the strains, as shown by others for morbilliviruses [31,26,20].

## Conclusions

Whatman® 3MM paper, although not specifically designed for nucleic acid preservation, is a logistically straightforward means of collection and preservation of specimens for further molecular studies. Adopting the non-invasive approach collection in combination with the paper matrix would permit early and frequent testing as well as providing a cost-effective source of samples to monitor PPRV infections in ruminants. The technology should be improved, however, to envisage isolating live PPRV field viruses from the same 3MM filter paper, which oppositely to the FTA card, retains the viability of the virus.

## Methods

### Ethics statement

Field studies were conducted in accordance with local legislation: Bengal Act N° I of 1920, The Bengal Cruelty to Animal Act, 1920. The study was conducted in animals in contact with outdoor environments with natural exposure to diseases (PPR is enzootic in Bangladesh). The tissues used in the study were ethically sourced. Blood and swabs were collected on live animals by aseptic means and/or by non-invasive methods, and tissues came from animals that died of infection or were humanely euthanized.

### Collection of samples from field cases using filter paper

PPR-suspected live animals were sampled at febrile and non-febrile stages. The blood samples were collected from jugular vein by aseptic means using sterilized 3 ml disposable syringes. Filter paper (Whatman® 3MM, Qualitative grade1), was cut into rectangular strips 5–6 cm long and 2.5-3 mm wide. A few drops of blood were poured on the paper from the base towards the tip, until it was completely soaked. Nasal swabs were collected with sterilized swab sticks, which were smeared on the filter paper. Filter papers were also smeared with the tissue homogenates. The filter paper loaded with sample was air dried avoiding direct sun light and was preserved in sterilized Eppendorf tube with proper labelling. The papers were transported to the laboratory without maintaining a cool environment and were stored at −70°C at the laboratory until analysis. Mesenteric and bronchial lymph nodes and lung tissues were also collected in sterile Falcon tubes at necropsy from PPR-suspected goats that died of infection. Collected mesenteric and bronchial lymph nodes were macerated using PBS at 20% (w/v) suspension and centrifuged at 3000 *g* for 10 min. The supernatant was collected in fresh sterile Falcon tubes and gentamycin was added at 500 μg/ml. Pairs of filter papers with smeared nasal swabs and with blood samples from the same animal were used in PCR tube directly as template RNA or kept at −70°C for viral stability study. When needed and for comparative purpose, RNA was extracted from the same tissue samples and from the vaccine virus, Nigeria 75–1 strain, using QIAGEN RNeasy Mini Kit as specified in the manufacturer’s instructions.

### Conventional Reverse Transcription PCR (RT-PCR) on Whatman® 3MM

The RT-PCR employed is adapted from methods described by Forsyth and Barrett [32] and Couacy-Hymann et al. [33]. Partial F genes (448 bp) and N (3′ 352 bp) were obtained by employing primers F1b and F2d (position: 760 > 784 and 1183 < 1207) and primers NP3 and NP4 (position: 1232 < 1255 and 1583 > 1560), respectively, and through a modification of the initial protocols. These were adapted to dried fluids spotted on Whatman® filter papers by using (1) a one-step method (OneStep RT-PCR mix, QIAGEN) and by (2) directly putting filter papers as source of RNA into PCR tubes without extraction of nucleic acid. RNase free water (33.5 μl) was added and tubes were heated at 95°C for 10 min, then immediately placed on ice. One-step protocol was then followed in a reaction mixture of 50 μl according to the manufacturer’s instructions and final amplicons were obtained at the expected length after 35 cycles. The field samples, 6–8 pieces of filter papers soaked with suspected blood or smeared with nasal swab, were tested for immediate and stability studies.

### Quantitative Real-Time Reverse Transcription PCR (qRT-PCR) on Whatman® 3MM

We have described a one-step real-time Taqman® RT-PCR assay (qRT-PCR) for PPRV targeting the nucleoprotein (N) gene to quantify PPRV [12]. This test was slightly modified to be transposable to Whatman® 3MM filter paper. It involved a first denaturation at 95°C for 10 min of pieces (2 mm^2^) of smeared filter papers in a tube containing 7.33 μl of ultrapure water and 12.5 μl of 2 × Kit Reaction buffer (AgPath.ID one-step RT-PCR kit, Applied Biosystem). Then PCR was performed in the same tube in a final reaction volume of 25 μl as described previously.

### Sequencing and phylogenetic analysis

PCR products amplified from filter papers smeared with nasal swab were purified with EZ-10 column DNA Gel Extraction Kit (Bio Basic, Inc., lot no. BS 353–080125) and were sequenced directly from a commercial source using the same primers. The N and the F gene sequences were deposited in GenBank under accession numbers HQ131961 and HQ898003, respectively and appear as the Bangladesh_2009 strain in the N and F trees.

With the Clustal W Program (www.ebi.ac.uk/clustalw), multiple sequence alignments of 252 and 279 nucleotides were generated according to sequence lengths available on GenBank for both the N and F genes, respectively. The RBOK wild strain of RPV and a measles virus strain were included as out-groups. From the alignments, phylogenetic reconstructions were done using a Bayesian analysis with the MrBayes program [34], version 3.2.1 (http://mrbayes.sourceforge.net/links.php). The sequence evolution model was proposed as HKY + I with 5 categories for the gamma distribution by Treefinder [35], version March 2011 (http://www.treefinder.de/). Consequently, the number of substitution types (Nst) was set at 2 and the Bayesian reconstruction was run until convergence between the two Monte-Carlo Markov Chains (MCMC) runs was attained as evidenced by an effective sample size for all posterior probabilities higher than 200 and a Potential Scale Reduction Factor (PSRF) used to compares the variance among runs with the variance within runs were all within 0.99-1.01. Trees were sampled every 1000 generations, the consensus tree was built up with a burn-in phase of 25%, and posterior probabilities were displayed as branch labels. Tree manipulations were done using the FigTree v1.4.0 program (http://tree.bio.ed.ac.uk/software/figtree/) and final edition was made under Adobe Illustrator CS3.
